# Comparing the effects of microwave radiation on 6-gingerol and 6-shogaol from ginger rhizomes (*Zingiber officinale* Rosc)

**DOI:** 10.1371/journal.pone.0214893

**Published:** 2019-06-10

**Authors:** Hui Teng, Kemueli T. Seuseu, Won-Young Lee, Lei Chen

**Affiliations:** 1 College of Food Science, Fujian Agriculture and Forestry University, Fuzhou, China; 2 School of Food Science and Bio-Technology, Kyungpook National University, Puk Gu, Daegu, Korea; Università degli Studi di Milano, ITALY

## Abstract

The active component obtained from ginger is a high value-added product, but continued research is required for improved extraction techniques that will lead to better quality extracts and greater yields. In this study, major functional compounds of 6-gingerol and 6-shogaol in ginger rhizomes (*Zingiber officinale Rosc*) were extracted using microwave assisted extraction (MAE). Possible ranges for optimal MAE conditions were predicted by merging of the contour plots of each response to observe the overlapping area of all responses. Optimal conditions predicted were ethanol concentration of 70%, extraction time of 10 min, and microwave power of 180 W. Verification tests carried out at a set of random condition within the above mentioned optimal ranges, which got experimental values for total soluble solid yield, antioxidant activity, 6-gingerol and 6-shogaol of 30.0±0.8%, 87.8±0.8%, 2.8±0.6 mg/g and 1.3±0.5 mg/g, respectively. Analysis results showed that steamed ginger sample contained lower 6-gingerol content, soluble solid as well as reduced antioxidant activity, but higher in 6-shogaol as compared with fresh sample.

## Introduction

Among the spices of the world, ginger assumes considerable importance, along with turmeric (*Curcuma longa* L.), as one of the most important and sought-after medicinal spices. Ginger, botanically known as *Zingiber officinale Rosc*, belongs to the family *Zingiberaceae* and in natural order *Scitamineae* [1]. Gingerhas been used as a spice for over 2000 years. Owing to its universal appeal, its spread has been rapid to both tropical and subtropical countries, from the China–India region, where ginger has been cultivated from time immemorial. It also was most valued for its medicinal properties, in ancient times, and also played a very important role in primary health care in India and China, and was widely used in European medicines as a carminative as well [2].

Recently, ginger has been increasingly used because of its low toxicity and its broad spectrum of biological and pharmacological applications[3,4]. Its roots contain polyphenol compounds (6-gingerol, 6-shogaol and its derivatives), which have a high antioxidant activity. Gingerols and shoagols which are phenolic ketones possess a wide range of pharmacological and physiological effects, including cardiovascular, gastro-intestinal (antiemetic, antinausea, antiulcer), antioxidant, anti-inflammatory, antimicrobial (analgesic, sedative, antipyretic, antibacterial), as well as thermogenic activities [5,6]. The shogaol is thought to be the dehydration products of the gingerols, derived from thermal processing (drying/heating) or long-term storage and are more pungent than the gingerol [7–9]. 6-Gingerol has been reported to exhibit antioxidative activity against linoleic acid autoxidation and peroxidation of phospholipid liposomes and to scavenge trichloromethylperoxyl- and 1, 1- diphenyl-2picrylhydrazyl (DPPH) radicals. In addition to these antioxidative effects, recent studies [10,11] revealed that it inhibits nitric oxide synthesis in activated J774.1 macrophages and prevents oxidation and nitration reactions induced by peroxynitrite, a strong reactive nitrogen species.

Microwave assisted extraction (MAE) introduces microwave to meet the increasing demands for new extraction techniques, amenable to automation with reduced solvent consumption and time, simplified manipulation and higher purity of final product[12]. Since the high temperatures reached by microwave heating reduces dramatically both the extraction time and the volume of solvent required, recoveries of analytes and reproducibility are improved and, therefore, MAE method should be considered as an interesting alternative with the limitation that experimental conditions must be chosen in order to avoid possible thermal degradation [13]. To control several variables in an experiment is challenging upon acquiring a maximum and quality optimum response. The disadvantage of conducting One Factor at Time (OFAT) experiment is failing to detect interaction between variables as the variables are not varied at one time. To overcome these challenges, response surface methodology (RSM) was developed, which is a combination of mathematical and statistical founded on the fit of a polynomial equation to experimental data, and can concurrently optimize the levels of independent variables to attain the best system performance [14,15].

In the present study, 6-gingerol and 6-shogaol were extracted from ginger rhizome using highly efficient MAE. The effects of three main independent variables including solvent, extraction time and microwave power and their levels on the quantity of the target dependent variables (6-gingerol, 6-shogaol, yield, and antioxidant activity) were investigated. Optimal conditions were predicted with the aid of response surface plot and overlapped counter plots according to the RSM. A comparison was also conducted between fresh freeze dried ginger and steamed freeze dried ginger in this study, inspecting the effects of freeze drying and steaming processes toward pungent by evaluating the contents 6-gingerol, 6-shogaol as well as antioxidant activity and total soluble solid yield.

## Materials and methods

### Sample preparation

About 5 kg of fresh ginger rhizomes were procured from a local Dongdaegu market in Daegu (W35°53′38′′, E128°39′38′′), South Korea. The fresh gingers were properly washed and were divided into two portions to undergo freeze drying process. The first portion was steamed for 18 h and the second remained fresh. Both portions were stored in the SAMWON Ultra Low Temperature Freezer for 24 hours at -20 degrees Celsius and then freeze dried in a SAMWON Freeze Dry System (Model No: SFDSF12, Samwon, Korea) at -70 degrees Celsius for 7 days. The freeze dried ginger were then ground to powder using an electrical blender, sieved through 40 mm mesh sieve and then sealed in a plastic bag and stored under 4 degrees Celsius for further use.

### Chemicals

1, 1-diphenyl-2-picryhydrazy (DPPH), 6-gingerol and 6-shogaol (HPLC standards,purity≥98%) chemicals were purchased from Sigma Chemicals Company in South Korea. Ethyl alcohol, methanol, petroleum ether, and acetone solvents of analytical grade were procured from Puksan, South Korea.

### Microwave assisted extraction procedure

Micro-digest (Soxwave 100, Prolabo, Fontenay, France) with a focused irradiation process under atmospheric pressure condition was used. The emission frequency of the extractor is 2450 MHz and microwave powers are linear and adjustable between 60 and 300 W with 30 W interval. The MAE device is equipped with a 250 mL quartz tube and a cool water circulation system of graham-type refrigerant column. Extraction process for freeze dried fresh ginger was as follows: 2 grams of powdered freeze dried fresh ginger was accurately weighed and placed into a 250 mL quartz tube. Fifty (50 mL) milliliters of solvent was added into the tube and inserted into the MAE machine where the MAE process occurs. Microwave power, time and solvent concentration were used concurrently as independent variables in the central composite design (CCD) application and were conducted as shown in Table 1. The microwave extracted sample was then filtered into a 100 mL conical flask using no.1 whatman filter paper, and volume up with the selected solvent to 100 mL mark. The filtrate was transferred into 100 mL sample bottle, sealed and refrigerated until further used.

**Table 1:** Central composite design matrix and experimental values of total yield extraction (TSSY), anti-oxidant activity content (AA), polyphenol compounds of 6-gingerol and 6-shogaol composed in fresh freeze dried ginger extract under microwave assisted extraction.

Exp. Run	Conditions			Response results			
	Ethanol (%)	Time (min)	Power (W)	TSSY (%)	AA (%)	6-gingerol (mg/g)	6-shogaol (mg/g)
1	25	6	120	24.04±0.05	46.43±0.47	1.582	0.280
2	25	6	240	22.72±0.35	44.45±0.36	1.653	0.279
3	25	12	120	24.53±0.20	46.59±0.44	1.739	0.284
4	25	12	240	25.72±0.12	44.81±0.48	1.964	0.271
5	75	6	120	24.16±0.21	53.49±0.23	2.850	1.563
6	75	6	240	24.08±0.29	51.67±0.29	2.767	1.495
7	75	12	120	23.33±0.12	55.74±0.52	2.833	1.645
8	75	12	240	25.65±0.09	52.33±0.46	2.868	1.645
9	0	9	180	14.77±0.17	46.43±0.29	0.951	*ND
10	100	9	180	13.04±0.14	91.16±0.53	2.585	1.248
11	50	3	180	27.79±0.12	44.38±0.76	2.687	1.282
12	50	15	180	26.53±0.12	41.24±0.52	2.928	1.743
13	50	9	60	28.37±0.20	44.46±1.43	3.246	1.529
14	50	9	300	26.77±0.09	37.44±0.46	2.882	1.441
15	50	9	180	28.21±0.17	44.46±0.35	2.867	1.559
16	50	9	180	27.79±0.17	44.50±0.29	2.932	1.601
17	50	9	180	26.51±0.17	44.61±0.68	3.053	1.640
18	50	9	180	28.40±0.16	44.38±0.89	3.149	1.662
19	50	9	180	26.99±0.23	44.46±1.36	2.946	1.742
20	50	9	180	27.84±0.14	44.50±0.26	3.150	1.832

*ND–not detected

### Total soluble solid yield (TSSY)

Total soluble solid extraction yield for freeze dried ginger was calculated as soluble solid content after evaporation to dryness of extract in a dish. Soluble solid content was analyzed according to the gravity method described by AOAC official method 922.10 [16]. Aluminum dish with diameter of 50 mm was dried in the oven for 1h at 105°C, cooled in a desecrator and then weighed (W_1_). Freeze dried ginger extract of 10 mL was pipette into the aluminum dish, and evaporated to dryness in the fume-hood by air drying. The dish was then allowed to dry completely in the oven at 105°C for 5 h, cooled in a desiccator for 10 minutes and weighed (W_2_). The process was done in triplicates.

$$\text{Total soluble solid yield extraction}\;\left(\text{\%}\right)=\frac{W_{2}-W_{1}}{W}\times 10\times 100$$

Where, W is the weight of ginger powder (g), W_1_ is the initial ginger extract weight before drying (g), W_2_ is the completely dried ginger extract weight (g)

### Antioxidant activity (AA)

The antioxidant activity of the ginger sample was determined by using DPPH assay [17] with some modification. DPPH reagent was prepared by mixing 0.0039 g DPPH powder to 100 ML pure ethanol and equilibrium for 2 h. Ginger extract of 100 μL was then mixed with 900 μL DPPH (100 μM) solution. The sample was shaken vigorously and kept in the dark at room temperature for 30 min. Control was prepared using pure ethanol. The absorbance was measured at 517 nm and the results were expressed according to the following equation:

Antioxidant ability (%) = (1 –absorbance of sample/absorbance of blank) x 100

### High performance liquid chromatography (HPLC) analysis of 6-gingerol and 6-shogaol

Two major functional components of ginger (6-gingerols and 6-shogaolshogaol) were isolated and identified using HPLC system with a UV detector (HPLC, JASCO International Co., Tokyo, Japan). About 2 mL extract was filtered through a 0.45 mm micro-filter (Millifilter, Milford, MA, USA) into a HPLC sample bottle. Separation was performed on an Xterra C18 reverse phase column (250 mm×4.6 mm, waters, USA) maintained at 30°C. The mobile phase consisted of methanol-water with ratio (65:35, v/v) was used. The HPLC operating parameters were as follows: injection volume, 60 mL; column flow rate of 0.8 mL/min; detection of absorbance was 280 nm. The 6-shogaol and 6-gingerol peaks were identified at 5.5 min and 7.8 min, respectively, by comparing their retention time to the individual standards (100 ppm 6-gingerol & 1000 ppm 6-shogaol standards). Quantitation was based on the molar absorption coefficient of the complex. Linearity was evaluated by obtaining calibration curves with multiple standards of the appropriate 6-shogaol and 6-gingerol in parallel with the samples. Standardization determination in seeds was based on the analysis of samples spiked with known quantities of 6-shogaol and 6-gingerol. The results were expressed as mg/g of dry wet basis of the ginger sample.

### Experimental design

In this study, single factor experiments by changing one factor at time while keep other factors at a constant level were employed to find suitable ranges for process variables under MAE. The optimization of MAE was conducted using central composite design (CCD) with 3 variables and 5 levels generated by built-in package (ADX module) of the SAS system (9.3 version, SAS institute, Cary, USA). The CCD matrices consist of 6 central points, 8 factorial points, and another 6 axis points at a distance of ±2 from the centre, resulting in 20 sets of experimental runs. Based on the primary experiment test using single factor experiment, ethanol was selected as suitable extraction medium, and microwave power, irradiation time and solvent were then selected as independent variables with ranges of 60–300 W, 1–13 min, and 0–100%, respectively. The process variables *X*_*i*_ was coded as x_i_ according to the equation below:

$$x_{i}=\left(X_{i}-\bar{X_{i}}\right)/\Delta X_{i}$$

where, x_i_ is the coded level, X_i_ is the natural level of the independent variable, $\bar{X_{i}}$ is the mean of the natural level of the independent variable, ΔX_i_ is the step change value.

Experimental data was then fitted into an empirical second order polynomial model using response surface regression analysis (RSREG) in SAS and presented in the following equation:

$$y=\beta_{o}+\;\sum \;\beta_{i}\;x_{i}+\;\sum \;\beta_{ii}\;x_{i}{}^{2}+\;\sum \;\beta_{ij}\;x_{i}\;x_{j}$$

Where, *Β*_*o*_ are the regression coefficients for intercept, *Β*_*i*_, are the regression coefficients for linear, *Β*_*ii*_ are the regression coefficients for quadratic, *Β*_*ij*_ are the regression coefficients for interaction terms, *x*_*i*_ and *x*_*j*_ are the independent variables. The CCD experiments were then executed accordingly in variable order. Fitness of model was estimated using analysis of variance (ANOVA), and determination coefficient R^2^ with the expression of its closeness to 1 indicates its suitability of prediction.

Experimental data were further processed using statistical three dimension surface and contour plots (StatSoft, Inc., Tulsa, OK, USA), from which the effects of variables to responses were evaluated. Merging of the contour plots of each response was constructed to observe the overlapping of each variable, thus indicating possible optimal conditions for all responses. The final evaluation was further validated on the optimal conditions of all responses.

### Statistical analysis

The analysis performed for TSSY and AA were in triplicates and results were expressed as means ± SD (standard deviations), 6-gingerol and 6-shogaol were directly analyzed using HPLC. Results obtained from the above responses were evaluated by using SAS software (Version 9.2, SAS Institute Inc, Cary, NC) with the level of statistical significant set at *p*<0.05 (95%).

## Results and discussions

There are a number of factors that should be considered when using microwave assisted extraction method such as the choice of solvent, microwave application time, microwave power, effect of contact sample surface area, and effect of temperature (Table 2). This is vital as it give us a fair idea of what sort of results to expect when running the main CCD experiment.

**Table 2:** Experimental variables and their levels in coded and un-coded forms for central composite design.

Independent variables	Symbols		Levels				
	Coded	Uncoded	-2	-1	0	1	2
Ethanol con. (%)	X_1_	x_1_	0	25	50	75	100
Extraction time (min)	X_2_	x_2_	3	6	9	12	15
Microwave power (W)	X_3_	x_3_	60	120	180	240	300

### The effect of solvents on microwave assisted extraction

The most important factor that affects MAE process is solvent selection. Choosing the right solvent will lead to a more efficient and effective extraction process. Based on the principles of like dissolves like and empirical extraction experience, five different solvents such as petrol ether, hexane, ethanol, ethyl acetate, and acetone were tested for ginger extraction. Results presented in S1 Fig showed the effect of five different solvents) tested with its effects on the responses of total soluble solid extracted yield, 6-gingerol and 6-shogaol under microwave assisted extraction, with a solvent to sample ratio of 25:1, Results showed that ethanol had the maximum extraction in TSSY (6.11%) and 6-gingerol (9.24 mg/g), but the maximum yield of 6-shogaol (17.44 mg/g) was from hexane extraction. The lowest extraction of TSSY (1.63%), 6-gignerol (2.15 mg/g) and 6-shogaol (1.64 mg/g) were produced using petroleum ether.

The capacity of the solvent to absorb microwave energy is high when the solvent presents high dielectric constant and dielectric loss [18]. [19,20]Both polar and nonpolar solvents can be used in MAE, and solvents like ethanol is sufficiently polar to be heated by microwave energy [21]. For the accessibility, eco-friendly and economic considerations, ethanol was chose as the proper extraction solvent.

### The effect of extraction time on MAE

Five extraction time variables ranging from 3–15 min, with microwave power of 180 W, 100% ethanol and solvent to sample ratio of 25:1 were used as conditions to test for the optimal range of irradiation time on the responses of TSSY, AA, 6-gingerol and 6-shogaol. In S2 Fig, results showed that the optimal extraction time for the four dependent variables of TSSY, AA, 6-gingerol and 6-shogaol were between 9–12 min. 6-gingerol and TSSY had the maximum extraction yield of 19.52 mg/g and 7.68%, respectively, at an irradiation time of 12 min, while AA and 6-shogaol produced the maximum yield of 91.26% and 29.81 mg/g respectively at 9 min.

The results indicated that time period of heating is an important factor that influences the ginger extraction process of MAE. The quantity of compounds extracted could increase with an increase in the extraction time, but there is an associated risk of degradation of 13 thermos labile components [22]. Previous studies showed that extraction times in MAE are very short compared to conventional techniques and usually vary from a few minutes to a half-hour, avoiding possible thermal degradation and oxidation, which is especially important for target compounds sensitive to overheating of the solute–solvent system [22,23]. Possible reason may be the irradiation time is influenced by the dielectric properties of the solvent. As indicated by Hu et al. [24], overheating occurs because of the high dielectric properties of the solvent, especially ethanol and methanol, and further dilution with water increases the heat capacity of the solvent combination [12], thus risking the future of 13 thermos labile constituents [19]. The dielectric properties of the solvent influence irradiation time optimization. Moreover, the overexposure to microwave radiation, even at low temperature or low operating power, was found to decrease the extraction yield because of the loss of chemical structure of the active compounds. The same behavior as shown in S1 Fig was also found and documented in previous studies [25–27].

### The effect of ethanol concentration on microwave assisted extraction

Previous studies have also shown that small amounts of water in the extracting solvent make possible the diffusion of water into the cells of the matrix, leading to better heating and thus facilitating the transport of compounds into the solvent at higher mass transfer rates. Thus, different ethanol concentrations were inspected in our further optimization study.

Data in S3 Fig showed the effect of ethanol concentration in MAE with 5 different percentages of ethanol (0, 25, 50, 75 & 100%). The fixed conditions for MAE were extraction time and microwave power at 9 min and 180 W respectively. Results showed that the optimal extraction for TSSY (21.76%) was obtained using 50% ethanol, 6-gingerol (6.80 mg/g) and 6-shogaol (1.36 mg/g) with 75% ethanol and AA (81.51%) with 100% ethanol. In many cases the extraction recovery is improved by the matrix moisture, which acts as a solvent. The moisture in the matrix is heated, evaporated, and generates internal pressure in the cell, which ruptures the cell to release the solutes, hence improving the extraction yield [28]. When increasing the polarity of the solvent, water addition has a positive effect on the microwave-absorbing ability and, therefore, facilitates the heating process [23,29]. Moreover, the additional water promotes hydrolyzation, thus reducing the risk of oxidation of the compounds [30].

### The effect of solvent to sample ratio of ethanol on MAE

Previous studies showed that the solvent-to-sample ratio was an important parameter to be optimized and also played an important role in extraction [31,32]. The solvent volume should be sufficient enough to immerse the plant matrix completely in the solvent throughout the entire irradiation process [19,20,29]. In case of MAE, a higher solvent: ratio of solvent to matrix ratio may not give better yield due to non-uniform distribution and exposure to microwaves [33].

Thus, the present study carried out different solvent to sample ratios (15:1, 20:1, 25:1, 30:1 and 35:1), which are shown in S4 Fig. Extraction time, microwave power and ethanol concentration were fixed at 9 min, 180 W and 100%, respectively. S4 Fig showed the optimal solvent to sample ratio for TSSY was 15:1 producing the highest extraction rate of 7.28%. The dependent variable decreased in quantity thereafter as the solvent increased to sample ratio 35:1 with a slight increase of TSSY. The solvent to sample ratio of 30:1 was optimal for AA with the maximum extraction rate of 79.36%, a gradual increase from 15:1 to this stage (30:1) after which it then decreased in AA quantity as the solvent increased to sample ratio of 35:1. However, it was found that the solvent to sample ratio of 20:1 was optimal for the main extraction targets (6-gingerol and 6-shogaol) in ginger, with the highest values of 18.37 mg/g and 29.56 mg/g, thus, the further study employed 20 as the optimal solvent to sample ratio.

### The effect of microwave power on MAE

Results in S5 Fig displayed the effect of the independent variable microwave power on the dependent variables TSSY and AA in ginger under MAE with a fixed solvent to sample ratio of 30:1, ethanol concentration of 100%, and extraction time at 9 min. TSSY with the highest extraction yield of 6.04% was attained at 180 W microwave power while AA and 6-gingerol with the highest yield of 81.15% and 19.64 mg/g were attained at 300 W microwave power. 6-shogaol however with the maximum extraction of 33.47 mg/g was produced at 240 W microwave power. Raner et al. [34] reported that variation of power from 500 to 1,000 W had no significant effect on the yield of flavonoids. The decrease in extraction yield was found at temperatures higher than 110°C because of instability of flavonoids and consequent thermal degradation [35]. In another case, higher microwave power led to thermal degradation of phenols when it was higher than 350 W (between 150 and 550 W) [26]. The possible explanation for the above mentioned results may because the microwave power is directly related to the quantity of sample and the extraction time required. However, the power provides localized heating in the sample, which acts as a driving force for MAE to destroy the plant matrix so that the solute can diffuse out and dissolve in the solvent. Therefore, increasing the power will generally improve the extraction yield and result in shorter extraction time [2,23,24,35]. In addition, when MAE is performed in closed vessels, the temperature may reach far above the boiling point of the solvent, leading to better extraction efficiency by the desorption of solutes from actives sites in the matrix [29]. On the other hand, high microwave power can cause poor extraction yield because of the degradation of thermally sensitive compounds. Also, rapid rupture of the cell wall takes place at a higher temperature when using higher powers, and as a result impurities can also be leached out into the solvent together with the desired solute [19,36,37]. Also Routray and Orsat [12] stated in their study that the efficiency increases with the increase in temperature until an optimum temperature is reached and then starts decreasing with the further increase in temperature: this happens because the selection of ideal extraction temperature is directly linked with the stability and, therefore, with the yield of the target compound. The factors microwave power and irradiation times influence each other to a great extent. In order to optimize a MAE procedure a combination of low or moderate power with longer exposure is generally selected.

### Optimization of Microwave assisted extraction for fresh freeze dried ginger

#### Fitting the model

According to the preliminary experiment results, Table 2 comprises of 3 variables and 5 stages of variables used for the optimization and execution of the central composite design (CCD). Under different conditions, freeze dried ginger powder was extracted using MAE at different ethanol concentrations, extraction times and microwave powers, ranging between 0–100%, 3–15 min and 60–300 W, respectively. Data produced from these experimental conditions were then fitted into a second order polynomial model, analyzed with regression analysis. Significances of the fitted models were assessed by using analysis of variance (ANOVA). The regression coefficients (Table 3) and ANOVA results (Table 4) for MAE of freeze dried ginger was also summarized, with the evaluation of three dimension surface and contour models effects between MAE process variables and responses of TSSY, AA, 6-gingerol and 6-shogaol. The R^2^ values for TSSY, AA, 6-gingerol and 6-shogaol are 0.9746, 0.9021, 0.8799 and 0.8126, respectively.

**Table 3:** Regression coefficient of predicted quadratic polynomial models of TSSY content, AA content, 6-gingerol and 6-shogaol content of fresh freeze dried ginger.

Coefficient	TSSY	AA	6-gingerol	6-shogaol
β_0_	18.749943^b)^	46.382727	-5.125795	-0.64625
Linear				
β_1_	0.542992	-0.68781	0.198339	0.0168
β_2_	-0.156214	0.720189	0.609754	0.04875
β_3_	-0.042411	0.077676	0.023904	0.002521
Quadratic				
β_11_	-0.005455	0.00975^a)^	-0.001082	-0.00011
β_21_	-0.004578	0.004	-0.002883	0
β_22_	-0.010677	-0.04471	-0.019141	-0.001944
Cross product				
β_31_	0.000198	-0.000123	-0.000138	-0.000005
β_32_	0.003408	-0.000972	-0.000326	-0.000041667
β_33_	0.000001989	-0.000241	-0.000035354	-0.000005208

**Table 4:** Fit statistics and numerical estimated level of extraction condition for the maximum TSSY, AA content, 6-gingerol and 6-shogaol content of the fresh freeze dried ginger.

Y_n_	R^2^	model *p*-value	x_1_	x_2_	x_3_	Max.	Morphology
TSSY	0.9746	<0.0001	49	13	267	28.77%	saddle point
AA	0.9021	0.0006	100	9	174	84.08%	saddle point
6-gingerol	0.8799	0.0015	73	11	180	2.75 mg/g	maximum
6-shogaol	0.8126	0.0110	73	11	180	1.35 mg/g	maximum

### The effect of process variables on the total soluble solid extraction yield (TSSY)

Table 2 presents a summary data, including the product of total soluble solid yield, which was verified with 20 different conditions of the independent variables under the CCD model. Test run 13&18 showed the maximum extraction TSSY value of 28.4% under the following conditions: 50% ethanol concentration, with irradiation time of 9 minutes and microwave power of 60 &180 W. Test run 10 showed the lowest TSSY value of 13.04% by MAE with 100% ethanol, 9 minutes extraction time and 180 W microwave power. The fitted model for TSSY as shown in the equation below was significantly (*p*<0.0001, R^2^ = 0.97) affected by the combination of ethanol concentration with time and ethanol concentration with power, but insignificant on the combination of microwave power with time. The values of the coefficient presented in Table 3 were used for the final predictive equation for TSSY and presented in the equation below:

$${\text{Y}}_{\text{TSSY}}=18.749943+0.542992{\text{X}}_{1}–\;0.156214{\text{X}}_{2}–\;0.042411{\text{X}}_{3}–\;0.005455{\text{X}}_{1}*{\text{X}}_{1}-0.004578{\text{X}}_{2}*{\text{X}}_{1}–\;0.010677{\text{X}}_{2}*{\text{X}}_{2}+\;0.000198{\text{X}}_{3}*{\text{X}}_{1}+\;0.003408{\text{X}}_{3}*{\text{X}}_{2}+0.000001989{\text{X}}_{3}*{\text{X}}_{3}$$

Where *X*_*1*_, *X*_*2*_ and *X*_*3*_ represent ethanol concentration, extraction time, and microwave power respectively. Based on the equation, three-dimensional response surface plots were constructed and shown in S6A and S6B Fig. These graphs depicted the impact of the three selected independent variables; ethanol concentration, extraction time and microwave power that determined the percentage extracted total yield under microwave extraction.

S6A Fig showed that at fixed ethanol concentration of 50%, optimal extraction of TSSY could be achieved between the range of 40 to 80 W microwave power and 3 to 5 min irradiation time. S6B Fig showed that at a fixed time of 9 min, the optimal extraction of TSSY could be obtained between 40 to 60% ethanol concentrations with no significant effect on microwave power. S6C Fig showed that a fixed power of 180 W, the optimal extraction of TSSY could be obtained at 4 to 16 min irradiation time and 40 to 60% ethanol.

Judge from Table 2, there was a huge drop in TSSY extraction (14.77, 13.04%) when the ethanol concentration was at its lowest (0%) and also at its maximum (100%). Increasing the irradiation time and microwave power to the maximum (15 min at 300 W) respectively showed no significant impact in TSSY extraction when ethanol was at 50% concentration. However, at 50% ethanol concentration, optimal TSSY extraction (26.5–28.4%) was achieved when microwave power ranges (60 & 180 W) and irradiation time range at 3 & 9 min were used. The results advocated that TSSY increased as ethanol concentration, extraction time, and microwave power increased, but only to a certain degree where further increases of the three independent variables affected the TSSY extraction to decline. This happened because the selection of ideal extraction temperature is directly linked with the stability and, therefore, with the yield of the target compound. On the other hand, high microwave power may cause poor extraction yield because of the degradation of thermally sensitive compounds. Also, rapid rupture of the cell wall takes place at a higher temperature when using higher power, and as a result impurities can also be leached out into the solvent together with the desired solute.

### The effect of process variables on 6-gingerol

The results of 6-gingerol, verified with 20 different conditions under the CCD model is presented in Table 2. The fitted model for 6-gingerol as shown in the equation below was significantly (*p*<0.05, R^2^ = 0.88, stationery point is a maximum) affected by extraction time and microwave power. The values of the coefficient presented in Table 2 were used for the final predictive equation for 6-gingerol and presented as:

$${\text{Y}}_{6-\text{g}}=\;-5.125795+0.198339{\text{X}}_{1}+\;0.609754{\text{X}}_{2}+\;0.023904{\text{X}}_{3}–\;0.001082{\text{X}}_{1}*{\text{X}}_{1}-0.002883{\text{X}}_{2}*{\text{X}}_{1}–\;0.019141{\text{X}}_{2}*{\text{X}}_{2}–\;0.000138{\text{X}}_{3}*{\text{X}}_{1}–\;0.000326{\text{X}}_{3}*{\text{X}}_{2}–\;0.000035354{\text{X}}_{3}*{\text{X}}_{3}$$

Based on the equation, surface and contour plots response in three-dimension were constructed and shown in S7A–S7C Fig. The effects of these three independent variables; ethanol concentration, extraction time and microwave power were evaluated and scrutinized. S7A Fig showed that at 50% ethanol concentration, optimum yield of 6-gingerol could be achieved either with a correlation between short irradiation time and high microwave power (2–5 min, 300 W) and or a correlation between long irradiation time and low microwave power (15 min, 40–80 W). S7B Fig showed that at a fixed time of 9 min, optimal extraction yield of 6-gingerol could be produced when microwave power is between 60–100 W and with ethanol concentration between 70–100%. S7C Fig showed a significant effect that at a fixed microwave power of 180 W, optimal extraction yield of 6-gingerol could be achieved when the extraction time is between 4–12 min and ethanol concentration between 50–90%.

### The effect of process variables on 6-shogaol

Data for 6-shogaol is shown in Table 2, tested under 20 different conditions with variation of ethanol concentration, extraction time and microwave power. The fitted model for 6-shogaol was significant with R^2^ value of 0.81 and *p* value of <0.05. As shown in the equation below, values of the coefficient data presented in Table 3 were fitted in and used for final predictive of 6-shogaol:

$${\text{Y}}_{6-\text{s}}=\;-0.646250\;+\;0.016800{\text{X}}_{1}+\;0.048750{\text{X}}_{2}+\;0.002521{\text{X}}_{3}–\;0.000110{\text{X}}_{1}*{\text{X}}_{1}+\;0{\text{X}}_{2}*{\text{X}}_{1}–\;0.001944{\text{X}}_{2}*{\text{X}}_{2}–\;0.000005{\text{X}}_{3}*{\text{X}}_{1}–\;0.000041667{\text{X}}_{3}*{\text{X}}_{2}–\;0.000005208{\text{X}}_{3}*{\text{X}}_{3}$$

S8A–S8C Fig are three dimension surface and contour plots responding to the independent variables and response of 6-shogaol which was further assessed and discussed. The highest yield of 6-shogaol was from test run 20 with the amount of 1.83 mg/g at 9 min extraction time, 50% ethanol concentration and microwave power of 180 W. Surface and contour plots of S8A Fig showed that the optimal extraction yield of 6-shogaol could be produced with an increase in extraction time, and with low to medium microwave power. As indicated in this plot to be between 40 and 180 W, and from 12 to 15 min irradiation time, when ethanol concentration was fixed at 50%. S8B Fig showed that maximum extraction yield of 6-shogaol could be achieved when ethanol concentration is between 60–90%, with microwave power between 100–220 W when irradiation time is fixed at 9 min. S8C Fig showed a similar trend as in S8B Fig with a good correlation that at a fixed microwave power of 180 W, optimal extraction yield of 6-gingerol could be produced when ethanol concentration is between 60–90% and with extraction time between 8–14 min. S8B and S8C Fig showed a good correlation between ethanol concentration, microwave power and extraction time. 6-shogaol increased as ethanol concentration, extraction time, and microwave power increased, but only to a certain degree where further increases of the three independent variables affected the 6-shogaol extraction to decline.

### The effect of process variables on antioxidant activity (AA)

Antioxidant activity percentage is presented in Table 2, obtained from fresh freeze dried ginger under MAE conditions. Table 3 presents regression coefficient of predicted quadratic polynomial models of experimental data. The fit statistics and numerical estimated level of extraction condition for the maximum AA as shown in Table 4 indicated that the stationary point was at a saddle point, with R^2^ of 0.90 and *p* value of <0.05. The fitted regression equation for MAE of antioxidant activity from ginger is shown as:

$${\text{Y}}_{\text{DPPH}}=\;46.355-0.687875{\text{X}}_{1}+0.700625{\text{X}}_{2}+0.07924{\text{X}}_{3}+0.009788{\text{X}}_{1}*{\text{X}}_{1}+0.004017{\text{X}}_{2}*{\text{X}}_{1}–\;0.042778{\text{X}}_{2}*{\text{X}}_{2}–\;0.000151{\text{X}}_{3}*{\text{X}}_{1}–\;0.001146{\text{X}}_{3}*{\text{X}}_{2}–\;0.000235{\text{X}}_{3}*{\text{X}}_{3}$$

Based on the equation above, antioxidant activity in ginger was plotted using three dimension surface designed, constructed from experimental data and showing effects on the interactions of three process variables under MAE. The three dimension surfaces plots showed that ethanol concentration and microwave power had great influence in AA yield compared to extraction time. Experimental run 1–4 showed that microwave power of 120 W had higher AA yield compared to that which were extracted at 240 W microwave power. Antioxidant activity increased further in yield from experiment run 5–8 when the ethanol concentration increased to 75% with a similar indication as in experiment run 1–4. This however dropped when ethanol concentration, extraction time and microwave power was made constant at 50%, 9 min and 180 W respectively. The least of the AA (41.2%, 37.4%) yield were produced when time and microwave power were both at its maximum that is, at 15 min and 300 W respectively. The highest AA yield of 91.2% was produce when 100% ethanol was used with 9 min extraction time and 180 W microwave power. Low ethanol concentration (0–25%) and high ethanol concentration (75–100%) produced more AA yield than 50% ethanol concentration. S9A Fig showed a significant effect, a good correlation between ethanol concentrations, extraction time and microwave power. At a fixed ethanol concentration of 50%, the optimal extraction yield for AA could be achieved with irradiation power between 110–210 W and irradiation time between 6–12 min. S9B and S9C Fig showed that microwave power and extraction time can still be increased further to get the maximum yield, however, there is no significant effect to indicate that the condition was optimal for maximum extraction. Based on the three figures, optimal conditions for AA was best illustrated in S9A Fig. This indicated that AA will increase with increase in time and power but only to its optimal point after which it will gradually subsided as time and microwave power increased.

### The optimization and verification process variables in MAE

The three MAE independent process variables namely ethanol concentration, extraction time and microwave power had been evaluated for a best combination ratio, to give an optimal condition for TSSY, 6-gingerol and 6-shogaol. In Table 4, results for TSSY showed that stationary point was at saddle point where as for 6-gingerol and 6-shogaol were at maximum point. Upon merging and overlapping concurrent optimized contour plots of these responses, a good correlation was shown between extraction time and ethanol concentration indicating a possible optimal condition. S10 Fig showed this combination within a specific area indicating to be the ideal condition. Table 5 recapitulate the optimal conditions captured from S10 Fig comprising of microwave power at 180 W, ethanol concentration between 60–80% and extraction time between 8–12 min. Experiments were conducted randomly within the vicinity of the projected ideal condition to verify its validity. Two sets of variation variables were used in the verification experiments including extraction time (9, 10, 11 min) and ethanol concentration (69, 70, 73%) while microwave power was fixed at 180 W. Results showed that from these randomly selected experimented values, extraction time of 10 min and ethanol concentration of 70% proved to be the most reliable condition. The predicted values presented in Table 5 included: 28% TSSY, 3.0 mg/g 6-gingerol, 1.0 mg/g 6-shogaol and 84.1% AA. The verification experimented values obtained for TSSY, 6-gingerol, 6-shogaol and AA were 30.0±0.8%, 2.8±0.6 mg/g, 1.3±0.5 mg/g and 87.8±0.8%, respectively. It can therefore be concluded that there is little variances between the predicted and experimented values, indicating that the fitted model for ginger extraction under MAE processing is satisfactory and reliable.

**Table 5:** Predicted optimal ranges, and forecasted experimental values of TSSY, 6-gingerol and 6-shogaol from freeze dried ginger under microwave-assisted extraction.

Process variables	Extraction time (min)	Ethanol concentration (%)
Predicted optimal ranges	8–12	60–80
Experimented optimal value	10	70
Responses	Predicted value	Experimented value
TSSY (%)	28.0	30.0±0.8
6-gingerol (mg/g)	3.0	2.8±0.6
6-shogaol (mg/g)	1.0	1.3±0.5
AA (%)	84.1	87.8±0.8

Ghasemzadeh et al. (2015) employed optimization work on the reflux extraction with different ethanol for 6-ginger and 6- shagol from ginger, which found the maximum values were 2.89 mg/g and 1.85 mg/g, respectively, and the maximum antioxidant activity was 84.3%, at the conditions of 78.9°C for 3.8 h. Subcritical water extraction was tried for ginger extraction as well (Anisa et al, 2014), which reported the highest 6-shagol content was 716.76 μg/g at 170°C, and elevated temperature showed hydrolysis effect of 6-gingerol to 6-shagol. These relevant studies confirmed the high efficiency of microwave-assisted extraction, which reduced the extraction time and protect its antioxidants from thermal destruction, and better for the ginger extraction.

### Comparison of TSSY, AA, 6-gingerol and 6-shgaol in fresh and steamed gingers

Comparisons tests between fresh and steamed gingers were conducted on TSSY, AA, 6-gingerol and 6-shogaol. The variable conditions used were at a fixed time of 10 min extraction, 180 W microwave power and 70% ethanol concentration. HPLC analysis peaks for 6-gingerol and 6-shogaol are shown on S11 Fig. Results in Table 6 showed that values of TSSY, AA, 6-gingerol and 6-shogaol in fresh ginger were 32.96%, 99.73%, 2.76 mg/g and 1.42 mg/g respectively and in steamed ginger, the values were 22.83%, 99.69%, 1.51 mg/g and 2.02 mg/g respectively. While comparing the data of the two samples, the content of TSSY, AA and 6-gingerol were found to be slightly higher in fresh freeze dried ginger, and 6-shogaol was found to be higher in steamed dried ginger. In Zancan et al. [38], it was reported that the antioxidant activity of the ginger extracts remained constant at ≈80% and decreased to ≈60% in the absence of gingerols and shogaols. Previous studies also indicated similar results as reported in previous studies [39–41] that gingerol content in fresh ginger rhizomes was higher than the solar dried ones. The reason was that gingerol is the main pungent principles in ginger rhizomes and thus become dehydrated at high temperature and as a result produces shagaol. This confirmed 6-gingerol’s high content in fresh ginger and 6-shogaol’s high content in steamed ginger shown in Table 6. The shogaol are thought to be the dehydration products of the gingerols, derived from thermal processing (drying/heating) or long-term storage [7–9] and are more pungent than the gingerols [5,38]. Also reported in Xiao et al. [35] that the steaming process could enhance the anticancer effects of ginger and that the increased level of shagoals contributed to the improved anticancer potential. A summary version of the previous studies on steaming effects is highlighted.

**Table 6:** Comparison between fresh freeze dried ginger and steamed freeze dried ginger.

Samples	Time (min)	Ethanol	Power (W)	TSSY	AA	6-gingerol	6-shogaol
		Conc. (%)		(%)	(%)	(mg/g)	(mg/g)
Fresh ginger	10	70	180	32.96±0.21	99.73±0.01	2.76	1.42
Steamed ginger				22.83±0.56	99.69±0.01	1.51	2.02

## Conclusion

The optimal microwave assisted extraction conditions for TSSY, 6-gingerol and 6-shogaol are the following conditions: ethanol concentration of 70%, extraction time of 10 min and microwave power of 180 W. The predicted values of the above conditions for TSSY, 6-gingerol, 6-shogaol and AA were 28%, 3.0 mg/g, 1.0 mg/g, and 84.1%, respectively. Results obtained from the verified experiment that was conducted using the above optimal conditions were as follows: 30.0±0.8% TSSY, 2.8±0.6 mg/g 6-gingerol, 1.3±0.5 mg/g 6-shogaol and 87.8±0.8% AA. Verification experimental data obtained under optimal MAE matched well with the predicted values.

Comparing the quantity of total soluble solid yield (TSSY), antioxidant activity (AA) and poly-phenolic compounds (6-gingerol and 6-shogaol) in fresh ginger and steamed ginger were experimented and results were obtained. The outcome of this experiment showed that while comparing the two sample extracts, TSSY, AA and 6-gingerol were shown to be high in fresh dried ginger and 6-shogaol was high in steamed dried ginger. On polyphenol compounds in ginger, previous study have indicated high content of gingerol in fresh ginger than in dried ginger. In addition, the high content of 6-shogaol in steamed ginger stated that shogaol is thought to be the dehydration products of the gingerols at high temperature from thermal processing (drying/heating) or long-term storage and as a result produces shagaol which is more pungent than gingerol.

## Supporting information

S1 Fig The effect of different solvents on total soluble solid yield (TSSY), 6-gingerol (6g), 6-shogaol (6s) from ginger rhizome under MAE (other factors were fixed at 5 min extraction time,180W microwave power, and solvent to sample ratio of 25:1). 6-Gingerol and 6-shogaol were on the dry weight of the extract, and each column represents the mean±SEM
(TIF)

S2 Fig The effect of MAE irradiation times on total soluble solid yield (TSSY), 6-gingerol (6g), 6-shogaol (6s), and antioxidant activity (AA) of ginger rhizome under MAE (other factors were fixed at microwave power of 180 W, 100% ethanol, and solvent to sample ratio of 25:1). 6-Gingerol and 6-shogaol were on the dry weight of the extract, and each column represents the mean±SEM.
(TIF)

S3 Fig The effect of different ethanol concentrations on total soluble solid yield (TSSY), 6-gingerol (6g), 6-shogaol (6s), and antioxidant activity (AA) of ginger rhizome under MAE ((other factors were fixed at 9 min extraction time, microwave power of 180 W, and solvent to sample ratio of 25:1). 6-Gingerol and 6-shogaol were on the dry weight of the extract, and each column represents the mean±SEM.
(TIF)

S4 Fig The effect of different ethanol to sample ratios on total soluble solid yield (TSSY), 6-gingerol (6g), 6-shogaol (6s), and antioxidant activity (AA) of ginger rhizome under MAE (other factors were fixed at 7 min extraction time, microwave power of 180 W, and 100% ethanol). 6-Gingerol and 6-shogaol were on the dry weight of the extract, and each column represents the mean±SEM
(TIF)

S5 Fig The effect of different microwave powers on total soluble solid yield (TSSY), 6-gingerol (6g), 6-shogaol (6s), and antioxidant activity (AA) of ginger rhizome under MAE (other factors were fixed at 9 min extraction time, 100% ethanol, and solvent to sample ratio of 25:1). 6-Gingerol and 6-shogaol were on the dry weight of the extract, and each column represents the mean±SEM
(TIF)

S6 Fig Three dimension surface and contour plots showing the relationship between (a) microwave power and extraction time, (b) microwave power and ethanol concentration, and (c) ethanol concentration and extraction time on total soluble solid extraction yield (TSSY) from ginger rhizome under MAE.
(TIF)

S7 Fig Three dimension surface and contour plots showing the relationship between (a) microwave power and extraction time, (b) microwave power and ethanol concentration, and (c) ethanol concentration and extraction time on 6-gingerol from ginger rhizome under MAE.
(TIF)

S8 Fig Three dimension surface and contour plots showing the relationship between (a) microwave power and extraction time, (b) microwave power and ethanol concentration, and (c) ethanol concentration and extraction time on 6-shagol from ginger rhizome under MAE.
(TIF)

S9 Fig Three dimension surface and contour plots showing the relationship between (a) microwave power and extraction time, (b) microwave power and ethanol concentration, and (c) ethanol concentration and extraction time on antioxidant from ginger rhizome under MAE.
(TIF)

S10 Fig Overlapped contour plot for the optimization of microwave-assisted extraction for total soluble solid yield, 6-gingerol and 6-shogaol from ginger rhizome. (TIF)

S11 Fig Comparison—HPLC profile of 6-gingerol & 6-shogaol in (a) fresh dried and (b) steamed dried ginger through microwave assisted extraction.
(TIF)
