# Supplementary figures and images for: Comparing the effects of microwave radiation on 6-gingerol and 6-shogaol from ginger rhizomes (Zingiber officinale Rosc)
Source: PLoS One. 2019 Jun 10;14(6):e0214893. doi: 10.1371/journal.pone.0214893 (PMC6557475; doi:10.1371/journal.pone.0214893)

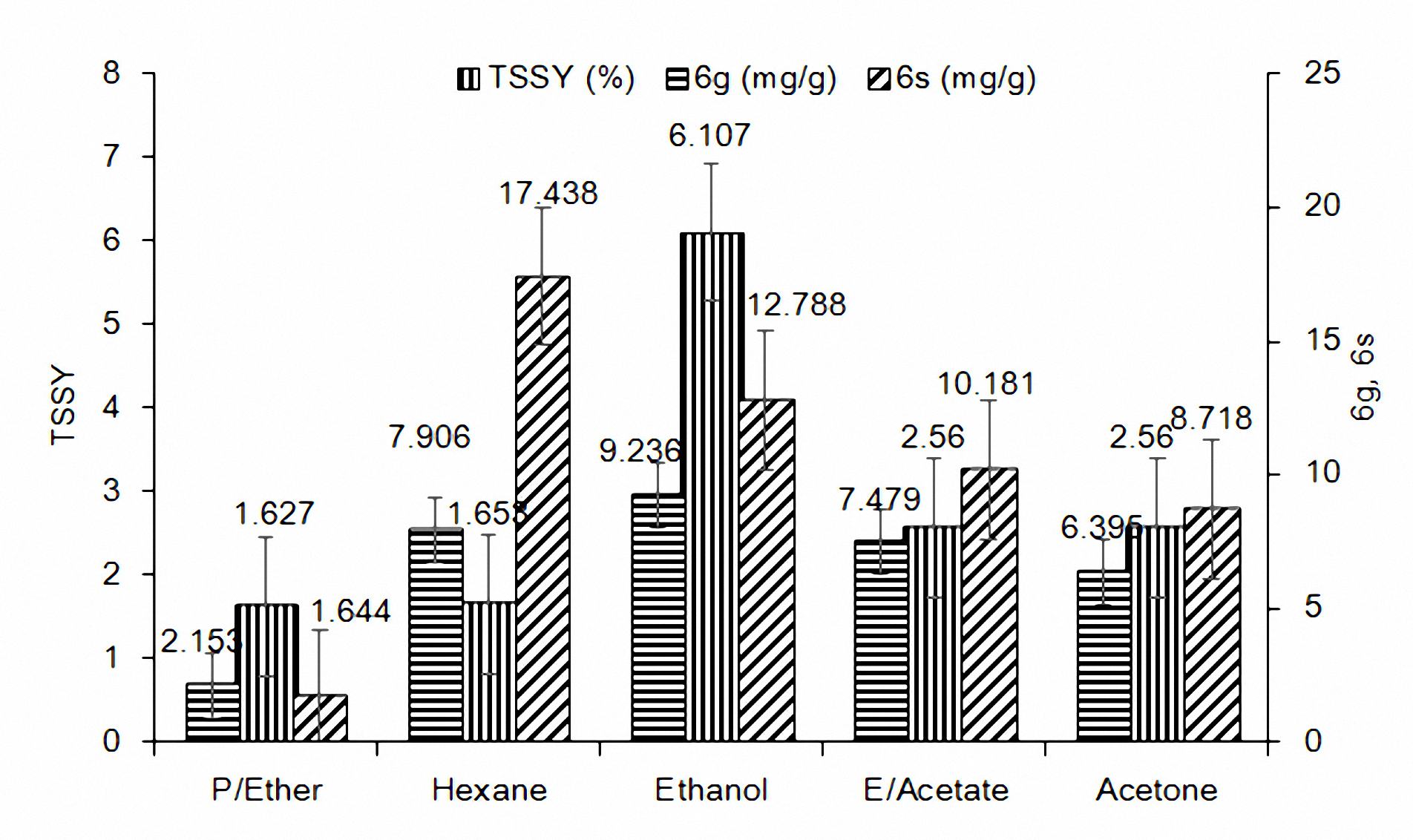

Supplement: S1 Fig — 6-Gingerol and 6-shogaol were on the dry weight of the extract, and each column represents the mean±SEM (TIF) [file pone.0214893.s001.tif]

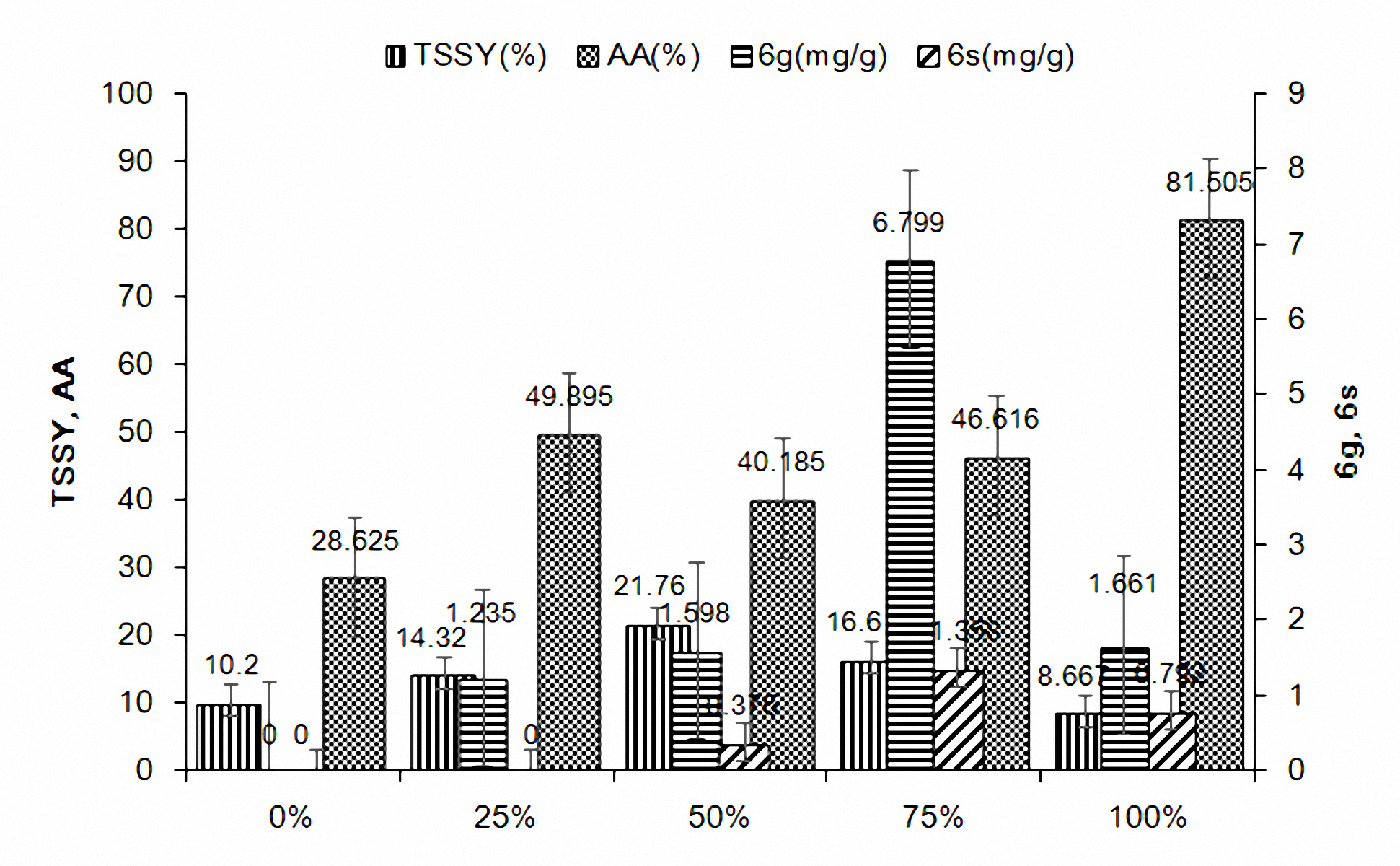

Supplement: S2 Fig — 6-Gingerol and 6-shogaol were on the dry weight of the extract, and each column represents the mean±SEM. (TIF) [file pone.0214893.s002.tif]

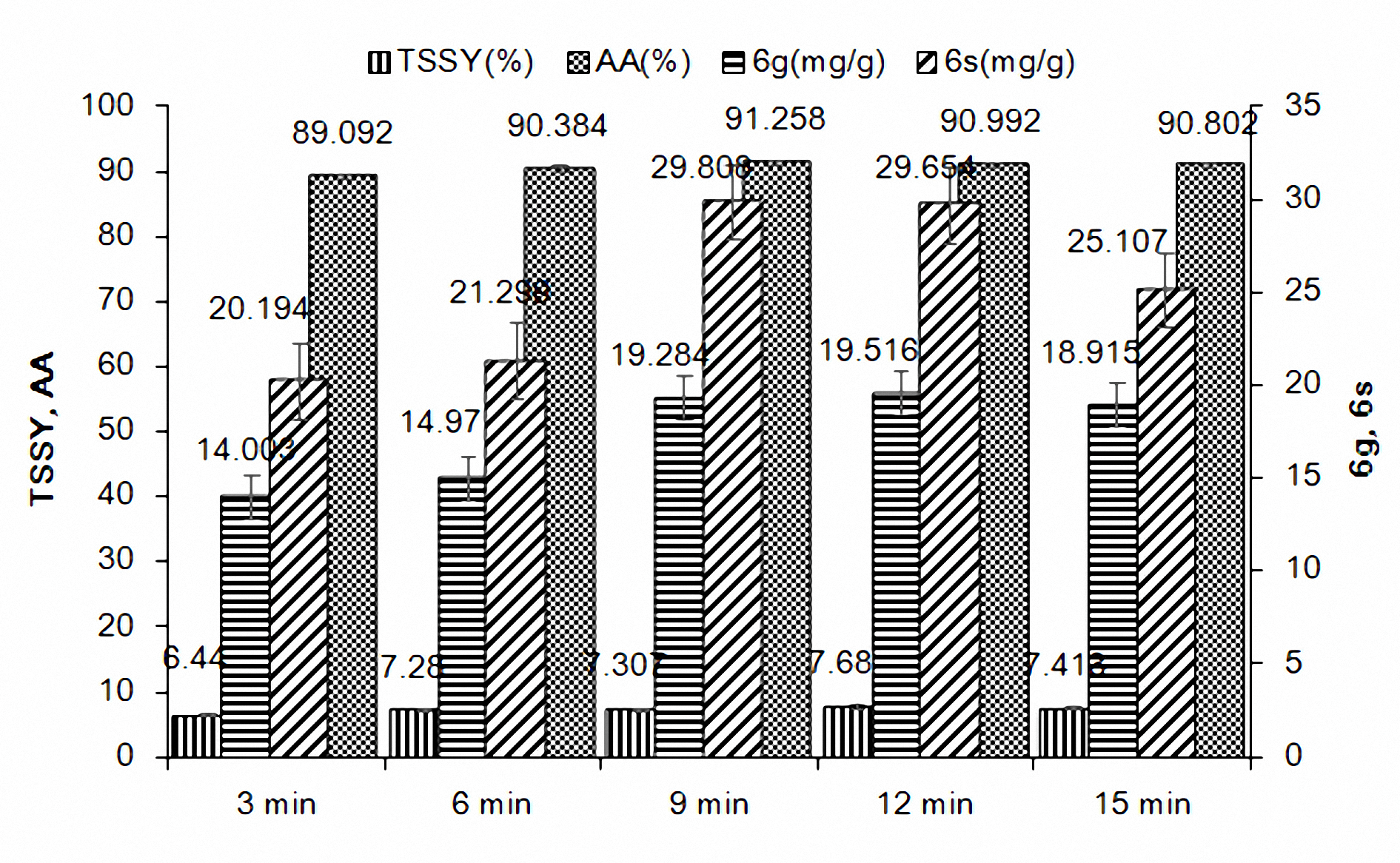

Supplement: S3 Fig — 6-Gingerol and 6-shogaol were on the dry weight of the extract, and each column represents the mean±SEM. (TIF) [file pone.0214893.s003.tif]

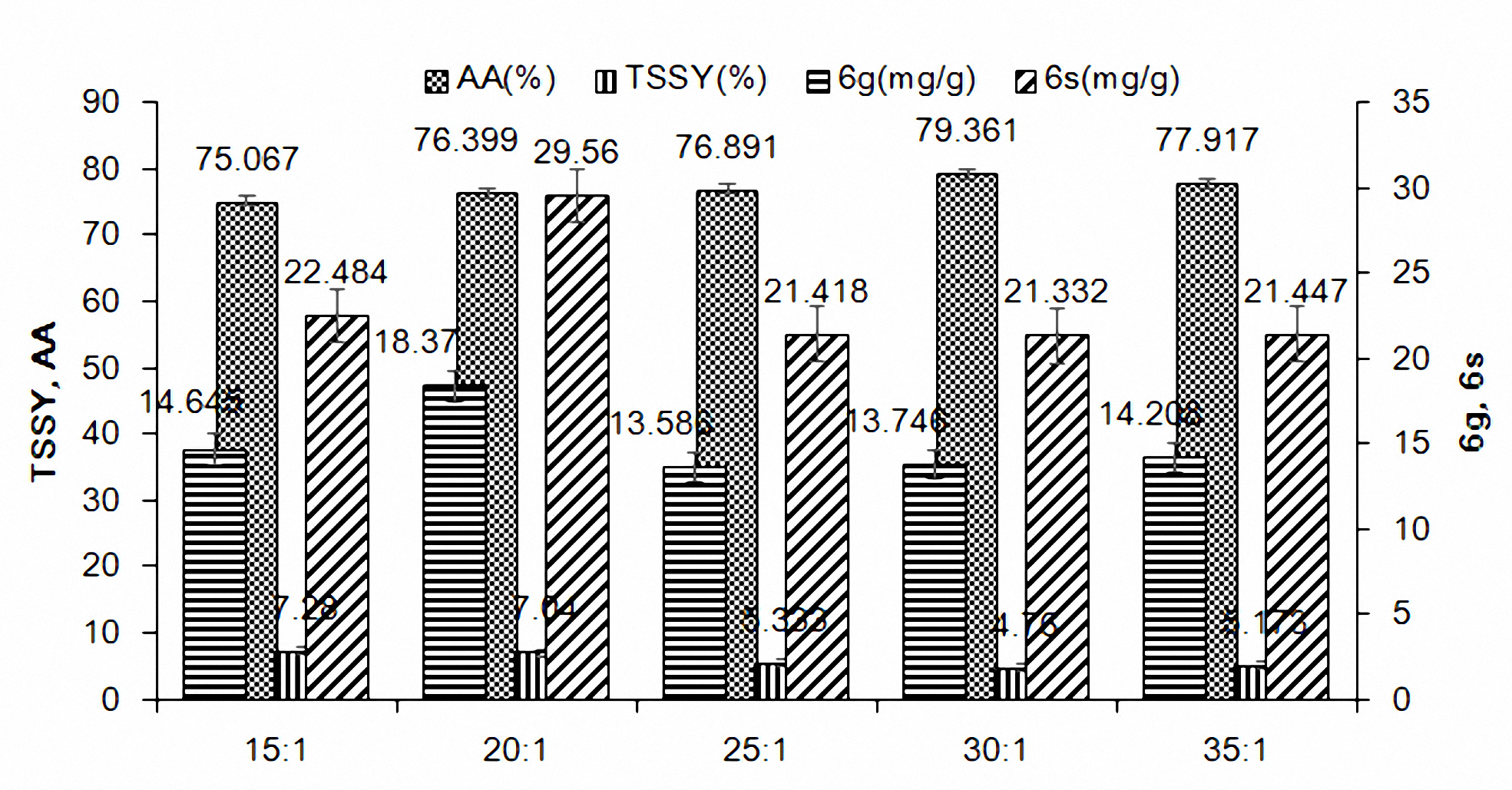

Supplement: S4 Fig — 6-Gingerol and 6-shogaol were on the dry weight of the extract, and each column represents the mean±SEM (TIF) [file pone.0214893.s004.tif]

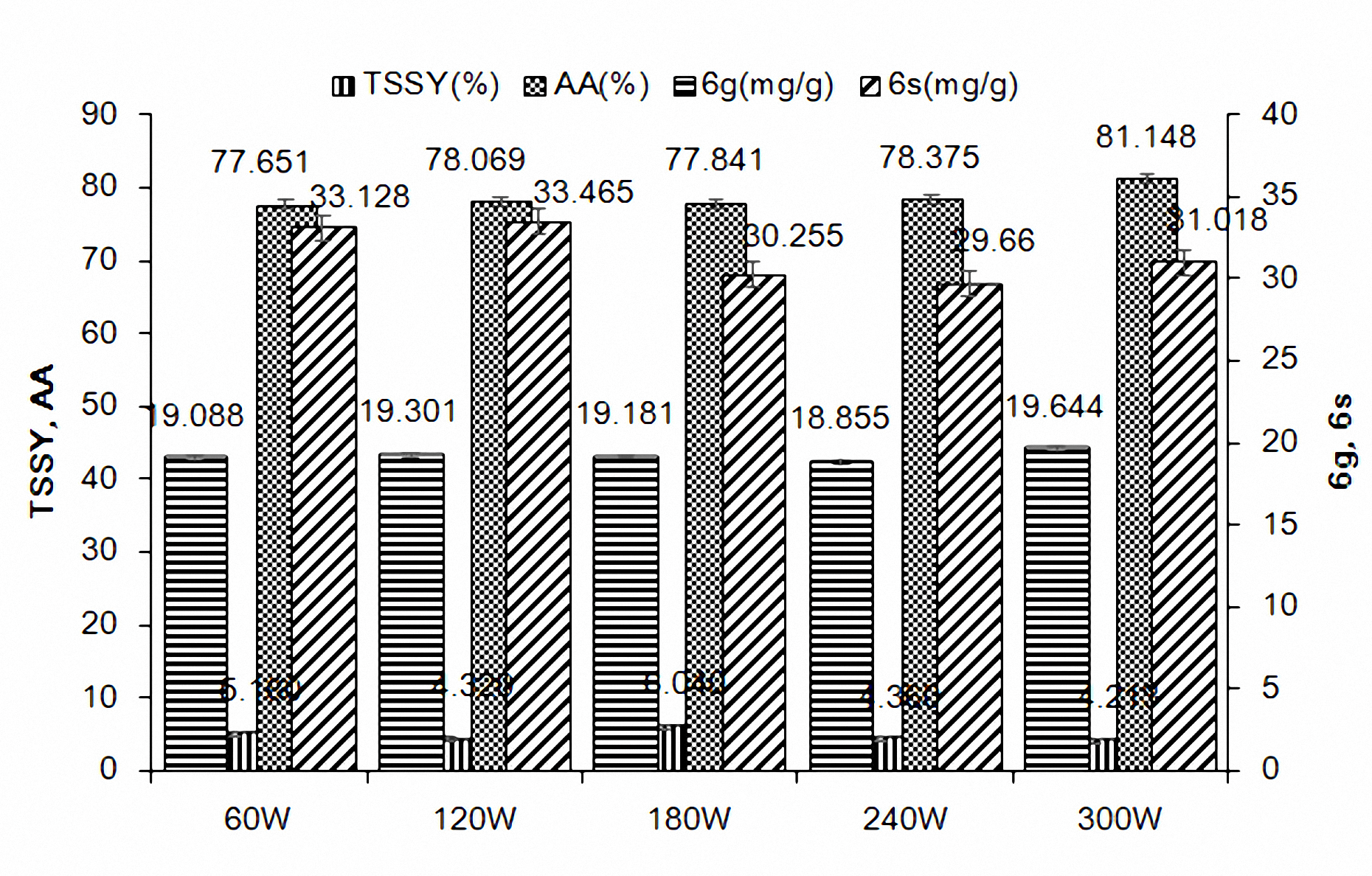

Supplement: S5 Fig — 6-Gingerol and 6-shogaol were on the dry weight of the extract, and each column represents the mean±SEM (TIF) [file pone.0214893.s005.tif]

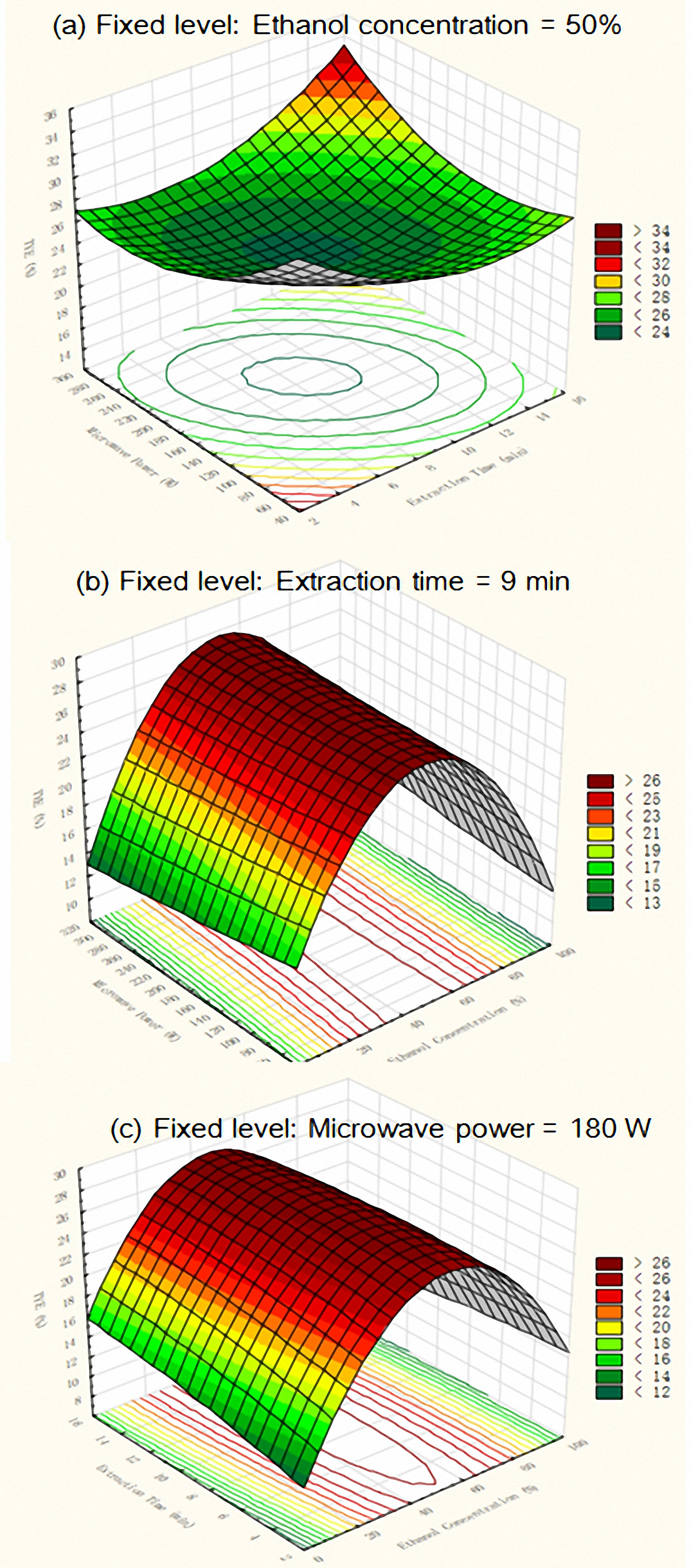

Supplement: S6 Fig — Three dimension surface and contour plots showing the relationship between (a) microwave power and extraction time, (b) microwave power and ethanol concentration, and (c) ethanol concentration and extraction time on total soluble solid extraction yield (TSSY) from ginger rhizome under MAE. (TIF) [file pone.0214893.s006.tif]

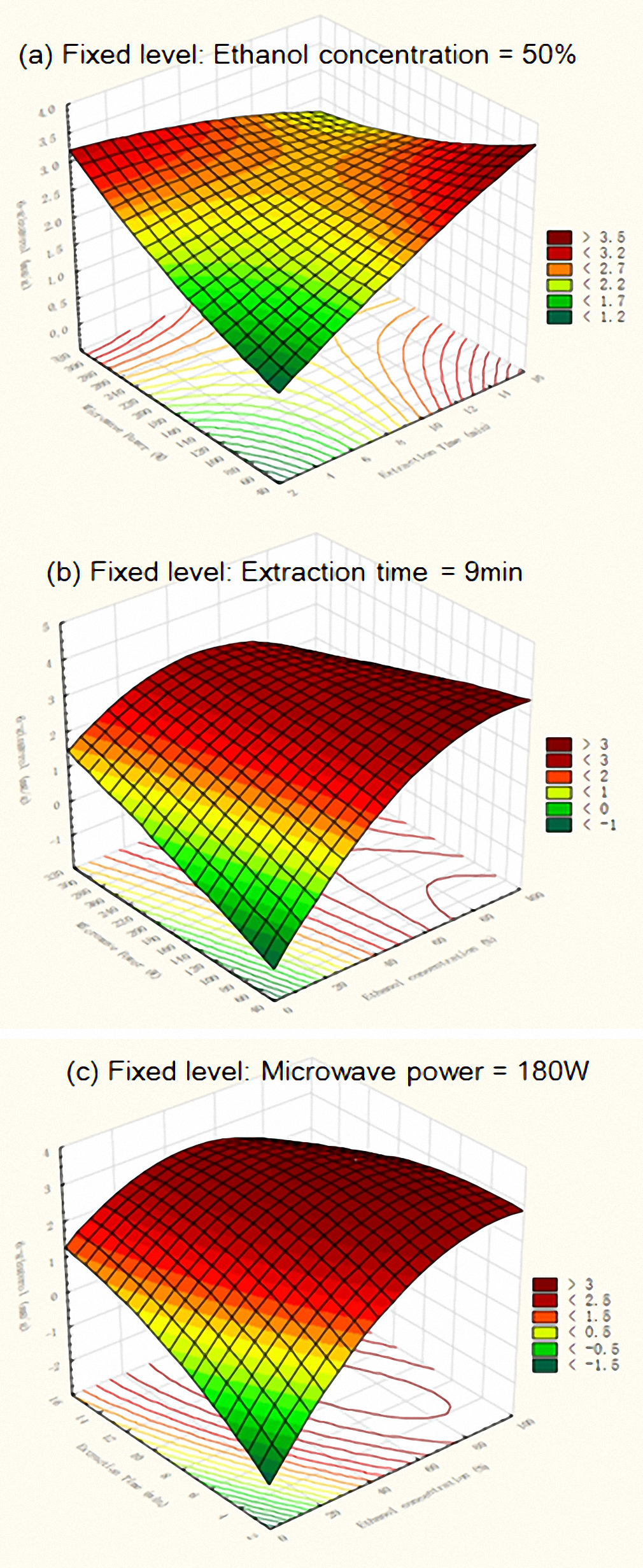

Supplement: S7 Fig — Three dimension surface and contour plots showing the relationship between (a) microwave power and extraction time, (b) microwave power and ethanol concentration, and (c) ethanol concentration and extraction time on 6-gingerol from ginger rhizome under MAE. (TIF) [file pone.0214893.s007.tif]

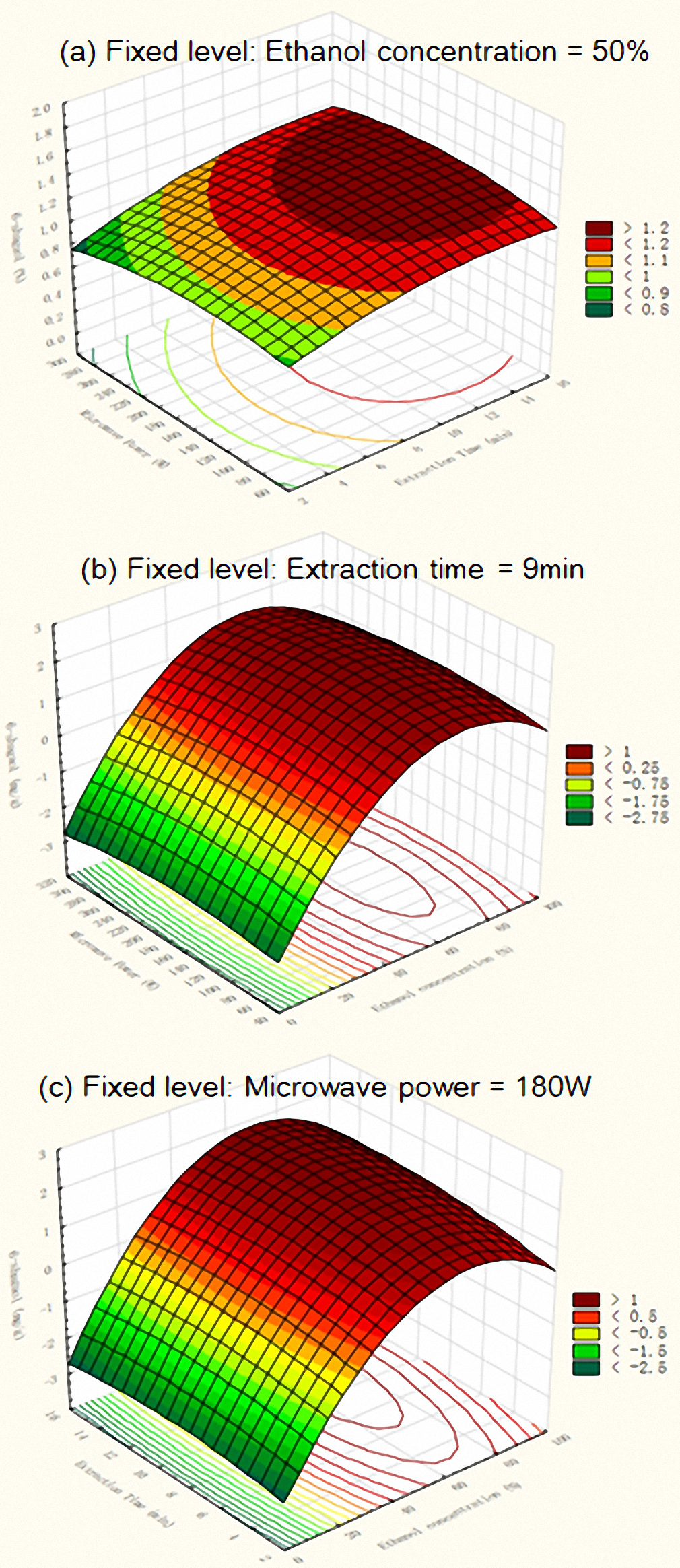

Supplement: S8 Fig — Three dimension surface and contour plots showing the relationship between (a) microwave power and extraction time, (b) microwave power and ethanol concentration, and (c) ethanol concentration and extraction time on 6-shagol from ginger rhizome under MAE. (TIF) [file pone.0214893.s008.tif]

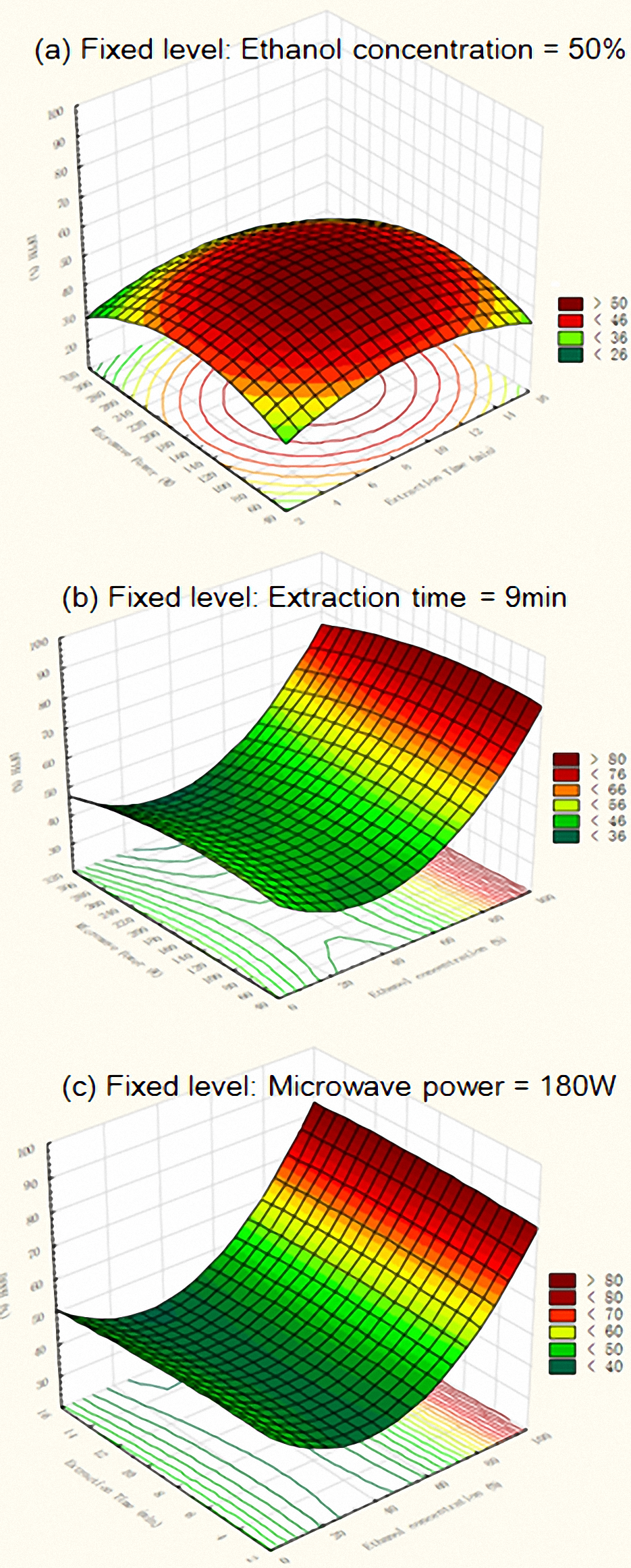

Supplement: S9 Fig — Three dimension surface and contour plots showing the relationship between (a) microwave power and extraction time, (b) microwave power and ethanol concentration, and (c) ethanol concentration and extraction time on antioxidant from ginger rhizome under MAE. (TIF) [file pone.0214893.s009.tif]

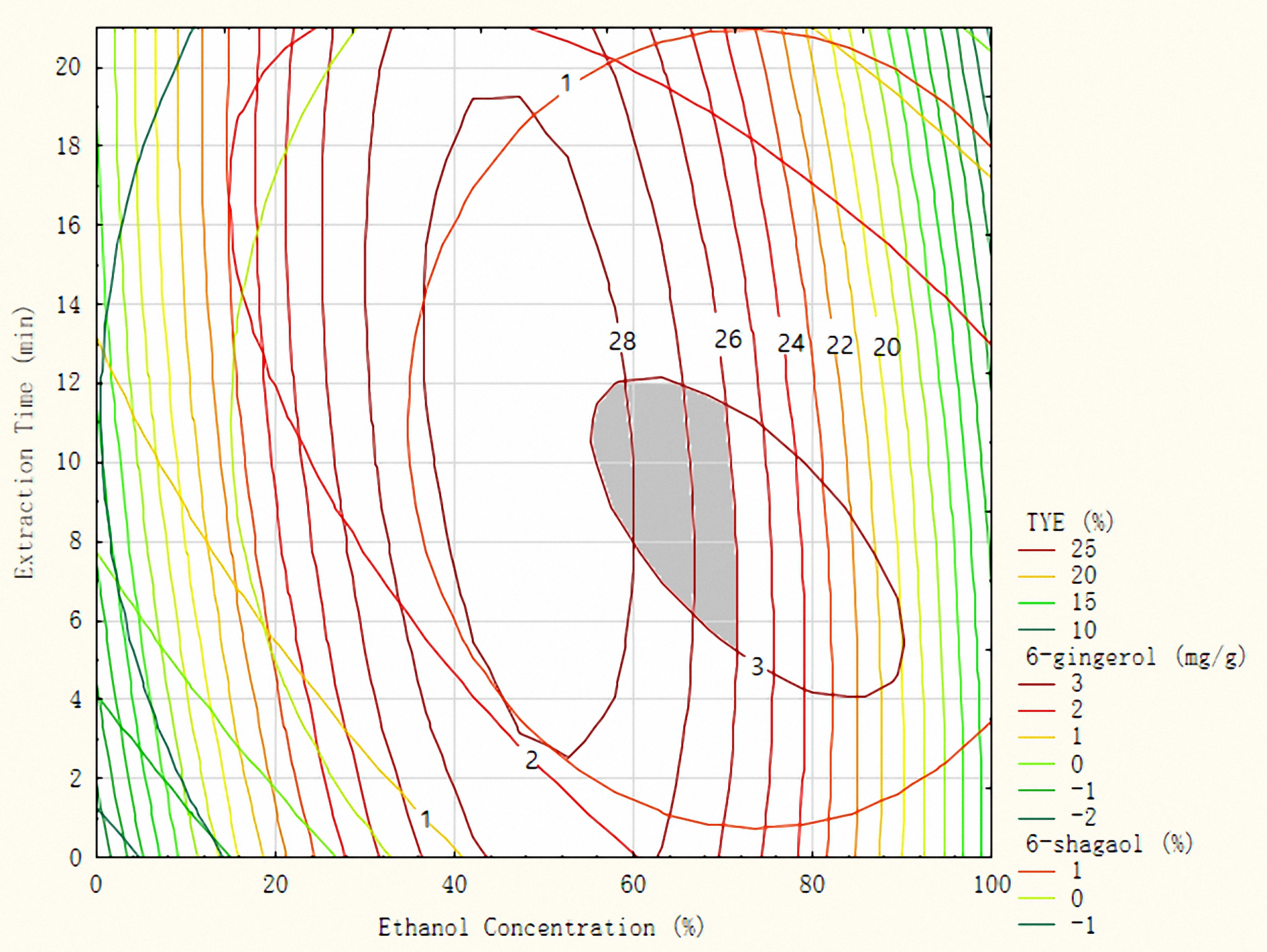

Supplement: S10 Fig — (TIF) [file pone.0214893.s010.tif]

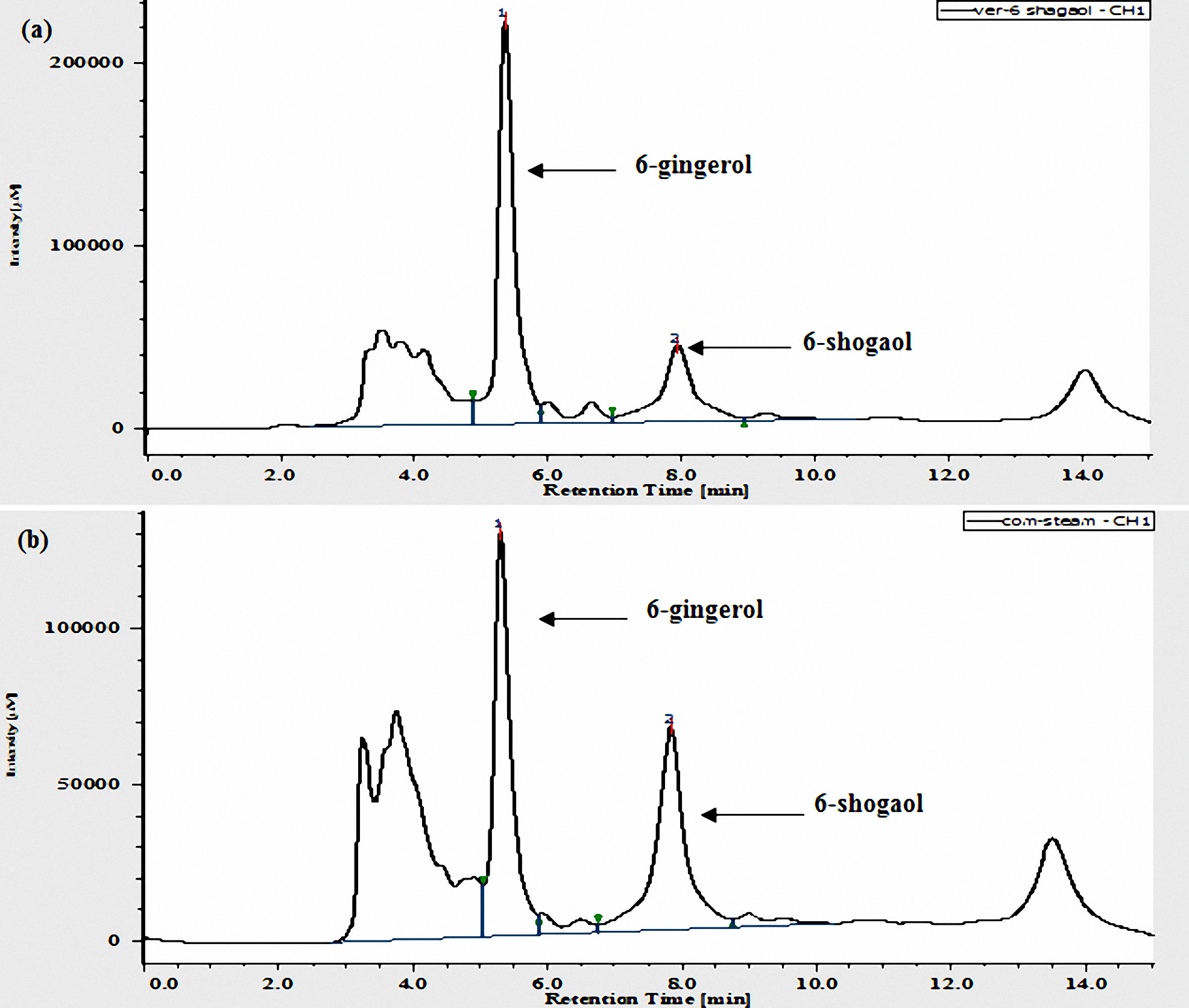

Supplement: S11 Fig — Comparison—HPLC profile of 6-gingerol & 6-shogaol in (a) fresh dried and (b) steamed dried ginger through microwave assisted extraction. (TIF) [file pone.0214893.s011.tif]
